# Prevalence and Factors Associated with the Risk of Eating Disorders Among Medical Students

**DOI:** 10.3390/healthcare14193314

**Published:** 2026-10-05

**Authors:** Wendy Vanessa Alfonso Lopez, Luz Patricia Ashton Yimes, Laura Lucia Quintana Espinosa, Hernan F. Guillen-Burgos, Milagros Isabel Roman Cohen, Alex Dominguez Vargaz, Henry J. Gonzalez-Torres

**Affiliations:** 1 Centro de Investigaciones en Ciencias de la Vida, Facultad de Ciencias de la Salud, Universidad Simón Bolívar, Barranquilla 080001, Colombia; wendy.alfonso@unisimon.edu.co (W.V.A.L.); luz.ashton@unisimon.edu.co (L.P.A.Y.); laura.quintana@unisimon.edu.co (L.L.Q.E.); hernan.guillenb@unisimon.edu.co (H.F.G.-B.); milagros.roman@unisimon.edu.co (M.I.R.C.); 2 Departamento de Psiquiatría y Salud Mental, Pontificia Universidad Javeriana, Bogotá 110111, Colombia; 3 Clinica de Nuestra Señora de la Paz, Orden Hospitalaria de San Juan de Dios, Center for Clinical and Translational Research, Bogota 110111, Colombia; aadominguez17@hotmail.com; 4 Data Analysis and Mining Department, Data & Project Consulting Service, Barranquilla 080001, Colombia

**Keywords:** eating disorders, medical students, mental health, EAT-26, risk factors

## Abstract

**Highlights:**

**What are the main findings?**

- One in five medical students (19%) screened positive for high risk of eating disorders on the EAT-26, with female sex (aOR = 3.03) and enrollment in the basic-science semesters (aOR = 2.79) as independent associated factors.
- Female students showed significantly higher Dieting and Oral Control scores than male students, while Bulimia scores did not differ by sex.

**What are the implications of the main findings?**

- Early, basic-science-stage medical students and women represent priority groups for routine EAT-26 screening and early mental health intervention within medical curricula.
- Restrictive eating behavior, rather than bulimic symptomatology, appears to be the predominant risk pattern in this population, pointing screening and prevention efforts toward dieting-focused rather than purging-focused indicators.

**Abstract:**

**Background/Objectives:** Eating disorders (EDs) represent a growing mental health concern in university populations, particularly among medical students, due to academic demands and psychosocial factors associated with their training. This study aimed to estimate the prevalence of symptoms suggestive of EDs and to analyze associated factors among undergraduate medical students during 2026. **Methods:** An observational, analytical, cross-sectional study was conducted among 167 medical students. Symptoms suggestive of EDs were assessed using the Eating Attitudes Test-26 (EAT-26), with a score ≥ 20 defining high risk. Descriptive and bivariate analyses were performed, along with a multivariate logistic regression model to identify associated factors. **Results:** The prevalence of high risk for EDs was 19% (n = 31). Women showed a higher prevalence of high risk compared to men (25% vs. 8.1%; *p* = 0.007), along with significantly higher scores in the Dieting (*p* = 0.014) and Oral Control (*p* = 0.007) domains. In the multivariate analysis, female sex (aOR = 3.03; 95% CI: 1.12–8.19; *p* = 0.029) and being enrolled in the basic-science semesters (I–III) (aOR = 2.79; 95% CI: 1.05–7.47; *p* = 0.041) were identified as independent associated factors. Co-occurring anxiety and depression was the most frequent psychiatric history among affected students. **Conclusions:** One in five students presented symptoms suggesting a high risk for EDs. Female sex and early-stage medical training were the main associated factors, identifying priority groups for early detection strategies and mental health promotion in the university setting.

## 1. Introduction

Eating disorders (EDs) constitute a major public health problem due to their increasing incidence, high disease burden, and close association with psychological disturbances, functional impairment, and premature mortality [1,2,3]. Although EDs have traditionally been studied in adolescents, recent evidence indicates that young adults, and particularly university populations, represent highly vulnerable groups for the development of risk eating behaviors [4,5,6].

Medical students are exposed to multiple factors that may favor the emergence of symptoms suggestive of EDs, including high academic demands, chronic stress, disrupted sleep habits, lifestyle changes, and an increased emotional burden associated with their training process [7,8,9]. Consequently, several international studies have reported prevalences of ED risk higher than those observed in the general population of the same age, suggesting that this population requires specific early detection and prevention strategies [10,11].

Among the associated factors, female sex has been consistently identified as one of the main predictors of dysfunctional eating behaviors. The greater sociocultural pressure related to body image, the internalization of thinness ideals, and body dissatisfaction contribute significantly to the increased risk in young women [12,13,14,15]. Likewise, the early years of university training have been described as a particularly vulnerable period due to the academic and psychosocial adaptation processes inherent to the transition into higher education [16,17,18].

Furthermore, EDs show a close relationship with other mental health disorders, especially anxiety and depression, conditions that frequently coexist and share common psychological and behavioral mechanisms [19,20,21]. Early identification of these associations is essential for developing preventive interventions aimed at preserving students’ psychological and academic well-being.

Despite growing international interest in this topic, the available evidence in Latin American medical students remains limited. In Colombia, specific studies on symptoms suggestive of EDs and their associated factors in this population are scarce, which hinders the implementation of screening and prevention programs based on local evidence. In this context, the present study aimed to estimate the prevalence of symptoms suggestive of eating disorders and to analyze their association with sociodemographic, academic, clinical, and lifestyle factors among undergraduate medical students at a university in Barranquilla, during 2026.

## 2. Materials and Methods

### 2.1. Study Design

We conducted an observational, analytical, and cross-sectional study among undergraduate medical students at a health sciences program in Barranquilla, Colombia, during 2026. The primary objective was to estimate the prevalence of symptoms suggestive of eating disorders (EDs) and to evaluate their association with sociodemographic, academic, clinical, and psychosocial factors. Risk for EDs was determined using the Eating Attitudes Test-26 (EAT-26) questionnaire.

### 2.2. Study Population

The study population consisted of undergraduate medical students older than 18 years. All students who voluntarily agreed to participate through electronic informed consent and completed the full battery of assessment instruments were eligible for inclusion. Exclusion criteria were: declining participation, age under 18 years, or incomplete responses to the survey instruments. A total of 167 students were included in the final analysis, of whom 31 were classified as high risk for EDs according to the EAT-26.

### 2.3. Sampling and Data Collection

A non-probabilistic convenience sampling method was used. The survey was distributed by institutional email to all enrolled undergraduate medical students; of approximately 1024 students enrolled in the program during the study period, 167 completed the survey (response rate ≈ 16.3%). Data were collected through an anonymous, self-administered electronic questionnaire distributed to eligible students, comprising sociodemographic, academic, clinical, and lifestyle variables in addition to the EAT-26 instrument. All participants provided electronic informed consent before completing the survey. Responses were stored in a secure institutional electronic database and subsequently anonymized prior to statistical analysis.

### 2.4. Definitions, Variables, and Instrument

The primary outcome was the presence of symptoms suggestive of EDs, defined as a score ≥20 on the EAT-26 [22]. The instrument comprises 26 items distributed across three validated subscales—Dieting, Bulimia and Food Preoccupation, and Oral Control—yielding total scores from 0 to 78 points; items are rated on a 6-point frequency scale (Always to Never) and scored 3-2-1-0-0-0, with one item reverse-scored, in accordance with the original validation criteria [23,24]. Independent variables included age, sex, marital status, academic semester, socioeconomic stratum, living arrangement, regular physical activity, sleep duration, perceived academic stress, and personal and family history of psychiatric disorders and EDs.

### 2.5. Statistical Analysis

Normality of continuous variables was assessed using the Kolmogorov–Smirnov test; given the significant departure from normality identified for the EAT-26 domain and total scores within each sex group (Shapiro–Wilk test, all *p* < 0.001), comparisons of these variables were performed using the Mann–Whitney U test rather than assuming normality. Continuous variables are presented as means with standard deviations (±SD), and categorical variables as absolute frequencies and percentages. Bivariate associations between categorical variables (sociodemographic characteristics, lifestyle habits, and clinical/psychiatric history) and risk classification (low vs. high) were assessed using Pearson’s chi-squared test or Fisher’s exact test, as appropriate to expected cell frequencies. Co-occurrence and clustering of psychiatric history were examined through an intersection (UpSet) analysis. Given the low number of high-risk events relative to the number of candidate predictors, factors independently associated with high risk for EDs were pre-specified a priori as sociodemographic and clinical variables with recognized relevance to eating disorder risk, and estimated using Firth’s penalized (bias-reduced) logistic regression, appropriate for this sample size; univariate and adjusted odds ratios (ORs) with 95% confidence intervals (CIs) were reported. As a sensitivity analysis, Firth’s penalized logistic regression including all originally considered candidate predictors was additionally performed (Table S1). A two-sided *p*-value < 0.05 was considered statistically significant. All analyses were performed using R statistical software (version 4.5.2; R Foundation for Statistical Computing, Vienna, Austria).

### 2.6. Ethical Considerations

The study was conducted in accordance with the ethical principles of the Declaration of Helsinki and Colombian national regulations for health research (Resolution 8430 of 1993, Ministry of Health). All participants provided electronic informed consent prior to inclusion, and data were anonymized to ensure confidentiality; researchers had no access to identifying information linked to individual responses. The study protocol was reviewed and approved by the Institutional Scientific Committee of Universidad Simón Bolívar (Minutes No. 171, 28 November 2025) and subsequently by the Research Ethics Committee (Approval Minutes No. 555, 2 December 2025; project code PRO-CEI-USB-CE-0633-00).

## 3. Results

### 3.1. Sociodemographic and Clinical Characteristics

The study included 167 undergraduate medical students, with a mean age of 19.9 ± 1.8 years (Table 1). The sample was predominantly composed of women (63%) and fourth- through sixth-semester students (47%). Regular physical activity was reported by 45% of participants, while a predominant proportion showed a borderline sleep pattern (63%) and medium-to-high perceived academic stress (96%).

Of the total sample, 31 students (19%) were classified as high risk for EDs (EAT-26 ≥ 20 points). Comparative analysis revealed statistically significant differences by biological sex, with a markedly higher proportion of women in the high-risk group compared with the low-risk group (84% vs. 58%; *p* = 0.007). Likewise, academic training stage showed a significant differential distribution (*p* = 0.014) (Figure 1); students in the basic-science semesters (I–III) accounted for 45% of all high-risk cases.

Regular physical activity was significantly less frequent among participants in the high-risk stratum compared with their low-risk counterparts (29% vs. 49%; *p* = 0.049). Age, marital status, socioeconomic stratum, family living arrangement, sleep duration, and perceived stress showed no statistically significant differences between groups (*p* > 0.05) (Table 1).

### 3.2. Medical and Psychiatric History

A family history of EDs was present in 12% of the sample. Although the proportion of students with this history was nearly twice as high in the high-risk stratum (19%) compared with the low-risk stratum (10%), this difference did not reach statistical significance (*p* = 0.212) (Table 2).

Regarding personal history, 16% of participants reported a prior diagnosis of a psychiatric disorder. Anxiety and depression were the most prevalent conditions, affecting 7.8% and 7.2% of the total population, respectively. Comparative analysis showed no statistically significant differences in psychiatric comorbidity burden or general medical history according to EAT-26 risk classification (all *p* > 0.05) (Table 2). Clustering analysis of psychiatric history revealed that isolated clinical anxiety (n = 4) and the simultaneous co-occurrence of anxiety and depression (n = 8) were the most frequent presentation phenotypes among students reporting a psychiatric history (Figure 2).

### 3.3. EAT-26 Test Evaluation

Assessment of symptoms suggestive of EDs using the EAT-26 questionnaire showed a mean overall score of 12.4 ± 9.9 points in the student cohort. Based on the standardized clinical cut-off, 19% of the university population was classified in the high-risk category for the development of EDs.

Stratified analysis by biological sex revealed a significantly greater impairment profile among women. The female cohort recorded higher total scores than their male counterparts (14.1 ± 10.9 vs. 9.5 ± 7.1; *p* = 0.005), with a threefold higher prevalence of high clinical risk in this subgroup (25% vs. 8.1%; *p* = 0.007). On the EAT-26 psychometric domains, women maintained significantly higher mean scores in the Dieting (*p* = 0.014) and Oral Control (*p* = 0.007) domains. In contrast, the Bulimia and food preoccupation domain showed no statistically significant differences between men and women (*p* = 0.6) (Table 3) (Figure 3).

Spearman correlation analysis assessed the bivariate relationship between EAT-26 domains, age, and perceived stress. Because the total EAT-26 score is the arithmetic sum of the Dieting, Bulimia, and Oral Control subscales, correlating each subscale directly with the total is subject to mathematical redundancy (part-whole correlation) and does not reflect an independent empirical relationship. To assess genuine inter-domain association, subscale–rest correlations were computed instead, correlating each subscale with the sum of the remaining two. These showed moderate, statistically significant positive correlations: Bulimia with the sum of Dieting and Oral Control (r = 0.51, *p* < 0.001), Dieting with the sum of Bulimia and Oral Control (r = 0.45, *p* < 0.001), and Oral Control with the sum of Dieting and Bulimia (r = 0.24, *p* = 0.001). In contrast, students’ chronological age and level of perceived stress showed no statistically significant correlations with any of its dimensions (*p* > 0.05) (Figure 4).

### 3.4. Factors Associated with High Risk of EDs

Given the low number of high-risk events relative to the number of candidate predictors, factors were pre-specified a priori as sociodemographic and clinical variables with recognized relevance to eating disorder risk, and estimated using Firth’s penalized (bias-reduced) logistic regression, appropriate for this sample size. Female biological sex was identified as a significant associated factor, with women showing approximately three times higher odds of high risk on the EAT-26 compared with men (aOR = 3.03; 95% CI: 1.12–8.19; *p* = 0.029) (Table 4).

Likewise, academic training stage proved to be an independent associated factor for high risk of EDs; being enrolled in the basic-science cycle (semesters I–III) nearly tripled the odds of symptomatology (aOR = 2.79; 95% CI: 1.05–7.47; *p* = 0.041) (Table 4, Figure 5). A sensitivity analysis including all originally considered candidate predictors, also estimated via Firth’s method, is reported (Table S1); the semester effect remained robust across specifications, while the sex effect attenuated when the model was saturated with additional covariates.

## 4. Discussion

The present study found that 19% of the medical students evaluated presented symptoms suggestive of high risk for eating disorders (EDs) according to the established EAT-26 cut-off, indicating that approximately one in five students could be at risk of developing clinically relevant eating behavior disturbances. This prevalence is consistent with recent studies conducted in university students, and particularly in medical students, where estimates range between 10% and 30%, depending on the instrument used, the sociocultural characteristics of the population, and the academic context evaluated [6,11,25]. A systematic review and meta-analysis published by Fekih-Romdhane et al. found that medical students show a higher frequency of ED risk than that observed in the general population of similar age, suggesting that the demands inherent to medical training constitute an environment conducive to the development of dysfunctional eating behaviors [10]. Our results provide local evidence confirming that this problem is also present among Colombian students and should be considered a priority within institutional mental health promotion strategies.

One of the most relevant findings was the independent association between female biological sex and high risk of EDs. Women showed a threefold higher prevalence than that observed in men (25% versus 8.1%). This finding is consistent with the international literature, which has consistently identified female sex as one of the main epidemiological determinants of EDs [1,12,15]. Several mechanisms have been proposed to explain this association, including greater internalization of thinness-centered aesthetic ideals, greater exposure to sociocultural pressures related to physical appearance, and higher levels of body dissatisfaction. Recent studies have shown that female university students more frequently display restrictive behaviors, weight concern, and maladaptive eating patterns, even in the absence of a formal psychiatric diagnosis [13,14]. Although the increase in EDs among men has been documented in recent years, the disease burden remains significantly greater among young women.

Analysis of the EAT-26 psychometric dimensions provides additional insight into the nature of this vulnerability. Female students showed significantly higher scores in the Dieting and Oral Control domains, while the Bulimia and food preoccupation domain remained relatively stable between both sexes. This pattern suggests that the predominant behaviors in this cohort are mainly related to voluntary food restriction and excessive intake control, rather than to compensatory behaviors or bulimic episodes. Similar findings have been described in university populations in Europe and Latin America, where restrictive behaviors constitute the most frequent initial manifestation within the spectrum of subclinical EDs [17,19,26]. From a preventive standpoint, these results are relevant because restrictive behaviors often precede the development of more severe clinical presentations, allowing for opportunities for early intervention before persistent pathological patterns become established.

Another finding of particular interest was the identification of students in the basic-science cycle (semesters I–III) as the most vulnerable group. After multivariate adjustment, being enrolled in the first semesters of the program nearly tripled the odds. This aligns with studies describing the transition into higher education as a critical period of psychological and social adaptation [16,18]. During this stage, multiple stressors converge, including changes in personal independence, new academic demands, modifications in eating habits, and disruptions to social support networks. Several authors have noted that the first university years represent a particularly susceptible time window for the development of mental health problems, including anxiety, depression, and eating disorders [20]. In medical students, these pressures may be amplified by the high level of academic competitiveness and the need to adapt to an intensive curriculum.

In the bivariate analysis, a lower frequency of regular physical activity was also observed among students classified as high risk for EDs. Although this variable lost significance in the multivariate model, the finding remains clinically relevant. Physical activity has been associated with better indicators of psychological well-being, emotional regulation, and positive body image perception [27,28]. Likewise, recent studies suggest that regular exercise practice may act as a protective factor against risk eating behaviors when performed for health purposes rather than exclusively for body weight control [29]. The absence of an independent association in our model could be related to sample size or to the influence of factors with greater explanatory weight, such as sex and academic level.

Regarding psychiatric history, although no statistically significant associations with ED risk were identified, clustering analysis revealed that the combination of anxiety and depression constituted the most frequent clinical phenotype among students with psychiatric history. This observation is in line with evidence describing a strong overlap between EDs and other affective disorders [2,30]. Anxiety and depression have been documented to share neurobiological, cognitive, and behavioral mechanisms with EDs, including alterations in emotional regulation, dysfunctional perfectionism, and avoidant coping styles. The coexistence of these conditions increases clinical severity and may hinder recovery if not identified in a timely manner. Although sample size limited the ability to demonstrate robust statistical associations, the observed frequency of this comorbidity supports the need to implement integrated mental health screening programs for university students.

From a psychometric standpoint, subscale–rest correlations indicated that Bulimia showed the strongest association with the remaining domains (r = 0.51), followed by Dieting (r = 0.45) and Oral Control (r = 0.24), while Bulimia was also the only domain that did not differ significantly between men and women, consistent with reports elsewhere that sex-based differences across EAT-26 subscales are domain-specific rather than uniform [31]. Taken together, these results indicate that dietary restriction and oral control are the domains driving the observed sex difference in this population; dietary restriction in particular has been widely described in university students and often represents the first observable manifestation of pathological concern about body weight and physical appearance [21]. In contrast, the absence of significant correlations with age or subjective perceived stress suggests that these factors, at least within the relatively homogeneous age range of the sample, have a smaller contribution compared with the sociocultural and academic determinants observed.

The findings of this research have important implications for institutional university well-being policies. The identification of particularly vulnerable groups, such as women and first-semester students, allows for targeted strategies for early detection, education on healthy eating habits, and strengthened psychological support. The implementation of periodic screening programs using validated instruments such as the EAT-26 could facilitate the early identification of at-risk students and help prevent progression toward clinically established disorders. In a context where the mental health of future healthcare professionals is a growing concern worldwide, the development of evidence-based preventive interventions represents an opportunity to promote healthier and more sustainable academic environments.

Limitations of the study include its cross-sectional design, which precludes establishing causal relationships; the use of a screening instrument rather than structured diagnostic interviews; and the fact that the study was conducted at a single institution, which could limit the generalizability of the results to the broader population of medical students. Additionally, the response rate (≈16.3%, consistent with typical rates for electronic surveys in university populations) does not rule out self-selection bias, as students more concerned about their eating habits may have been more likely to participate. The relatively small number of high-risk cases (n = 31) constrains the statistical power available for multivariate modeling; to mitigate this, the regression analysis was restricted to a small set of pre-specified predictors and estimated using Firth’s penalized logistic regression, with a full-model sensitivity analysis reported (Table S1), but estimates should still be interpreted with caution. Nevertheless, the use of a bias-reduced modeling approach, the use of a widely validated instrument, and the simultaneous evaluation of multiple clinical, academic, and psychosocial factors strengthen the validity of the findings obtained. Future multicenter and longitudinal studies will allow for a better understanding of the temporal evolution of these symptoms and the mechanisms involved in their development.

## 5. Conclusions

One in five medical students presented symptoms suggestive of high risk for eating disorders. Female biological sex and enrollment in the first semesters of medical training were the main independent associated factors, while female students showed higher levels of dietary restriction and oral control of intake. These findings identify a specific vulnerability profile within the student population and highlight the importance of incorporating early detection strategies and mental health promotion efforts targeted at higher-risk groups during medical training.

## Figures and Tables

**Figure 1:**
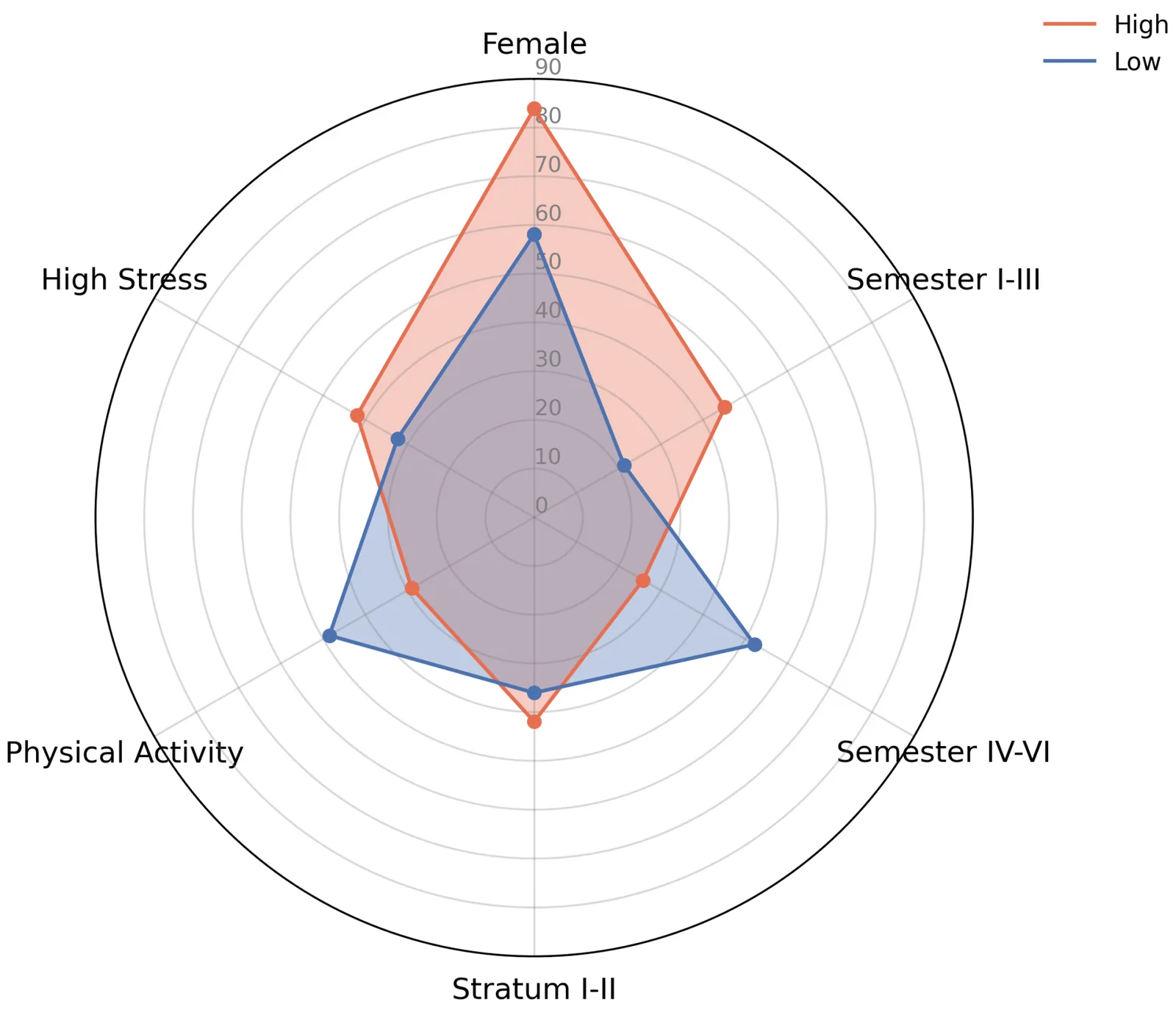
Sociodemographic and clinical profile by EAT-26 risk classification. Axes show the percentage of students with each characteristic, by high-risk (red) and low-risk (blue) group.

**Figure 2:**
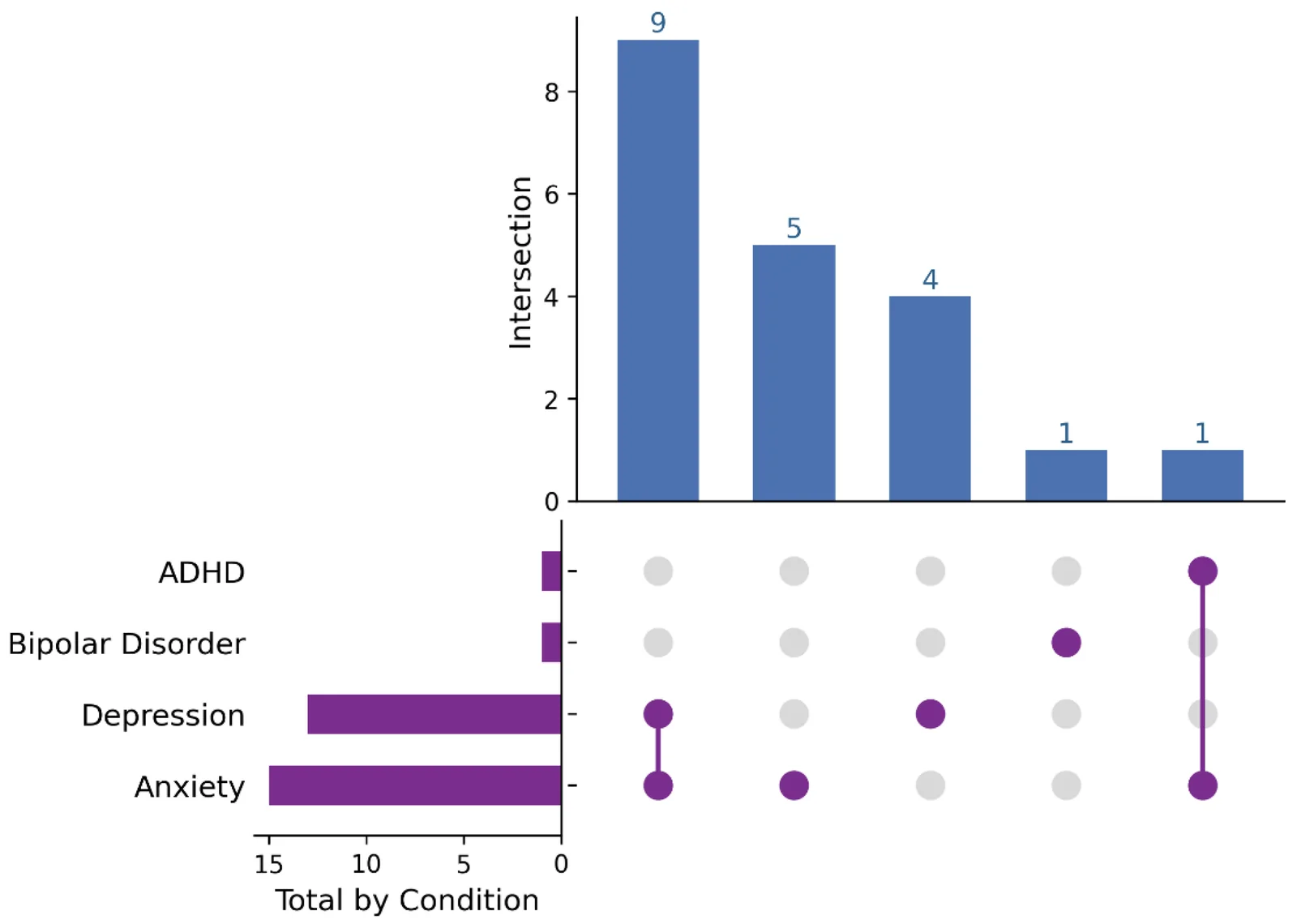
UpSet plot of psychiatric history co-occurrence. Horizontal bars show total frequency per condition; vertical bars show the number of students with each combination of conditions (nodes below). ADHD: Attention-Deficit/Hyperactivity Disorder.

**Figure 3:**
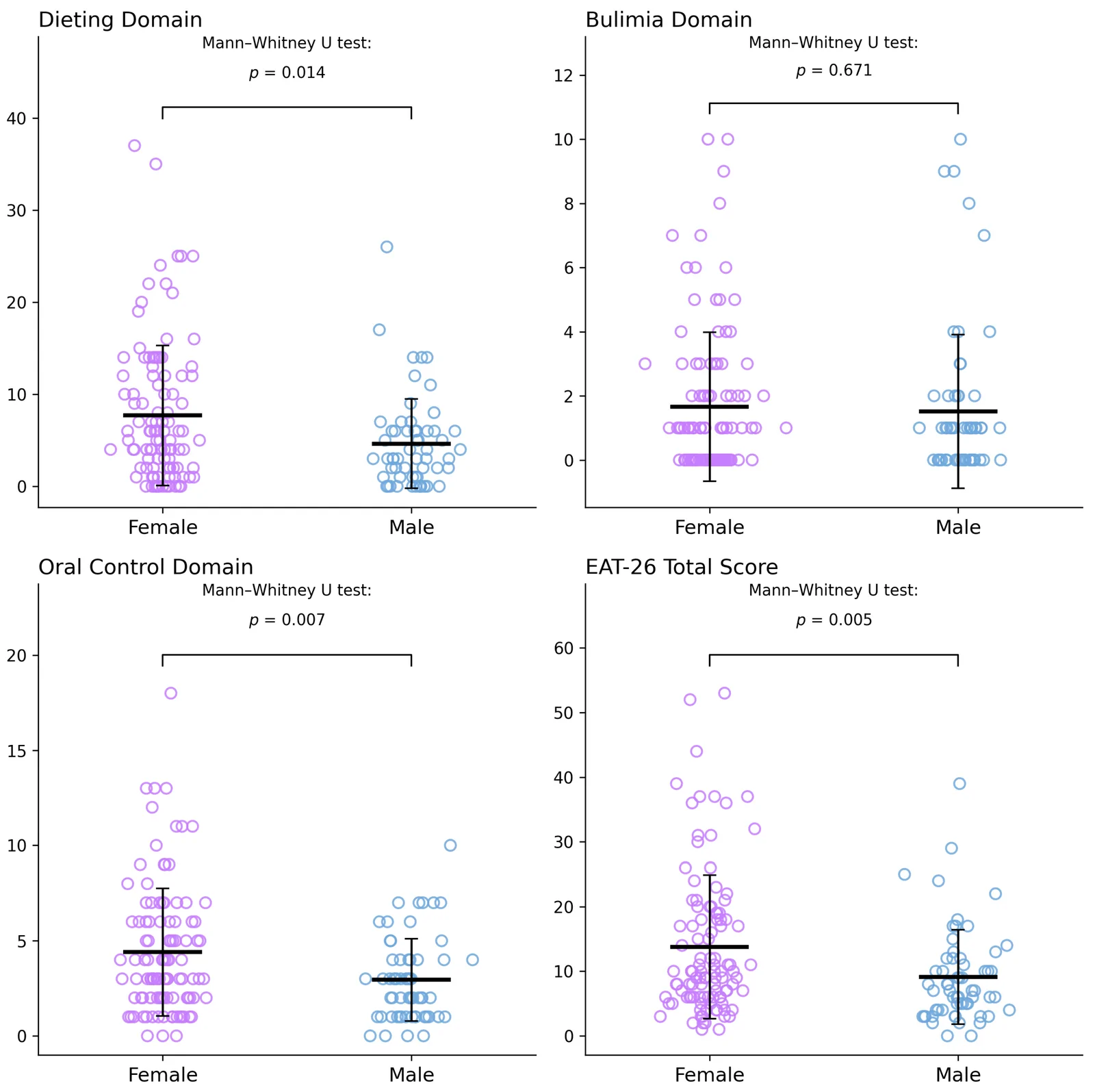
Distribution of EAT-26 total and domain scores (Dieting, Bulimia, Oral Control) by sex. Horizontal lines show the mean ± SD; *p*-values are from the Mann–Whitney U test.

**Figure 4:**
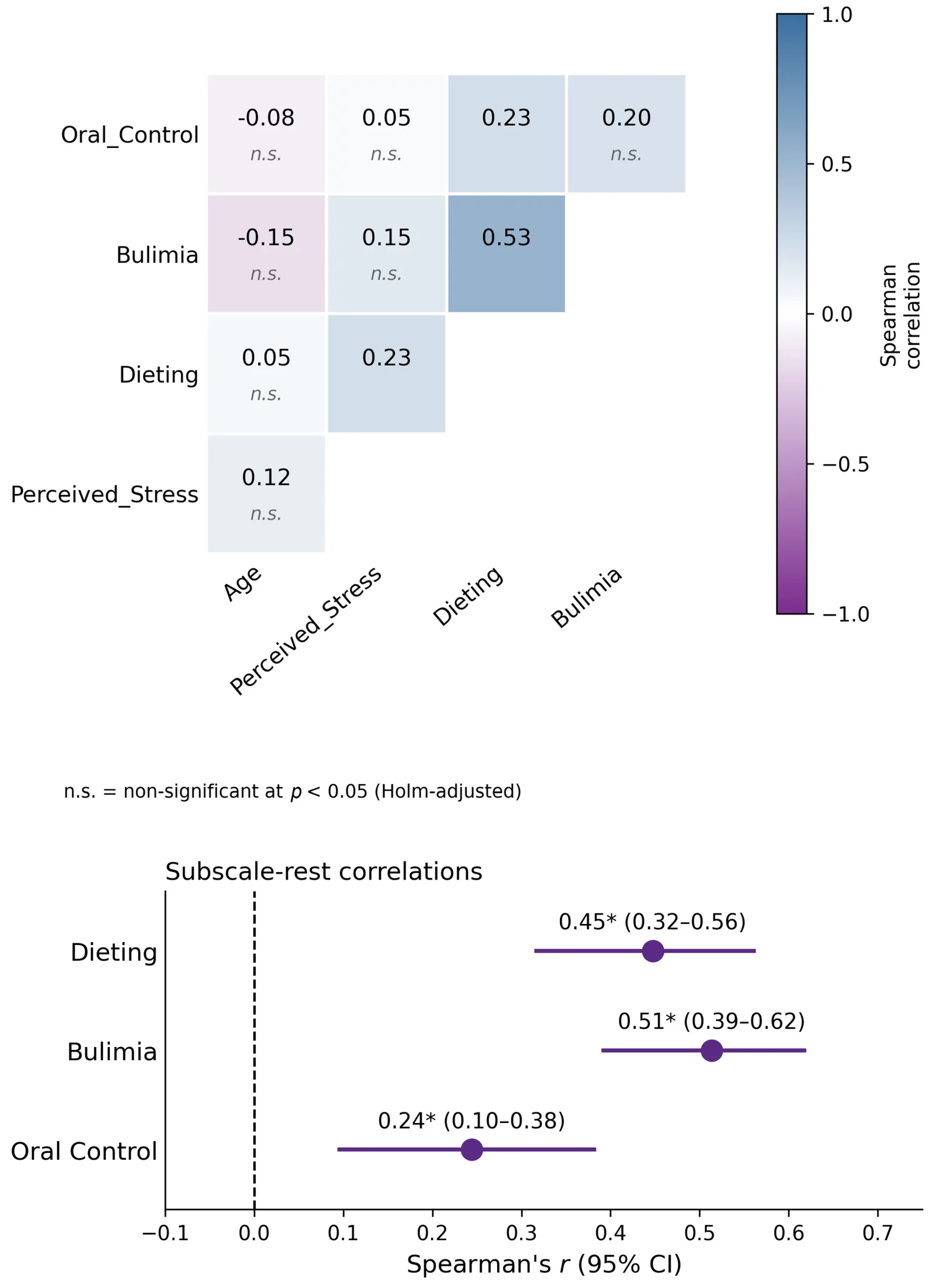
(**Top**) Spearman correlation matrix (EAT-26 domains, age, perceived stress); values are rho, “n.s.” = non-significant after Holm’s adjustment. (**Bottom**) ubscale-rest correlations (points = r, bars = 95% CI; * *p* < 0.05); dashed line marks r = 0.

**Figure 5:**
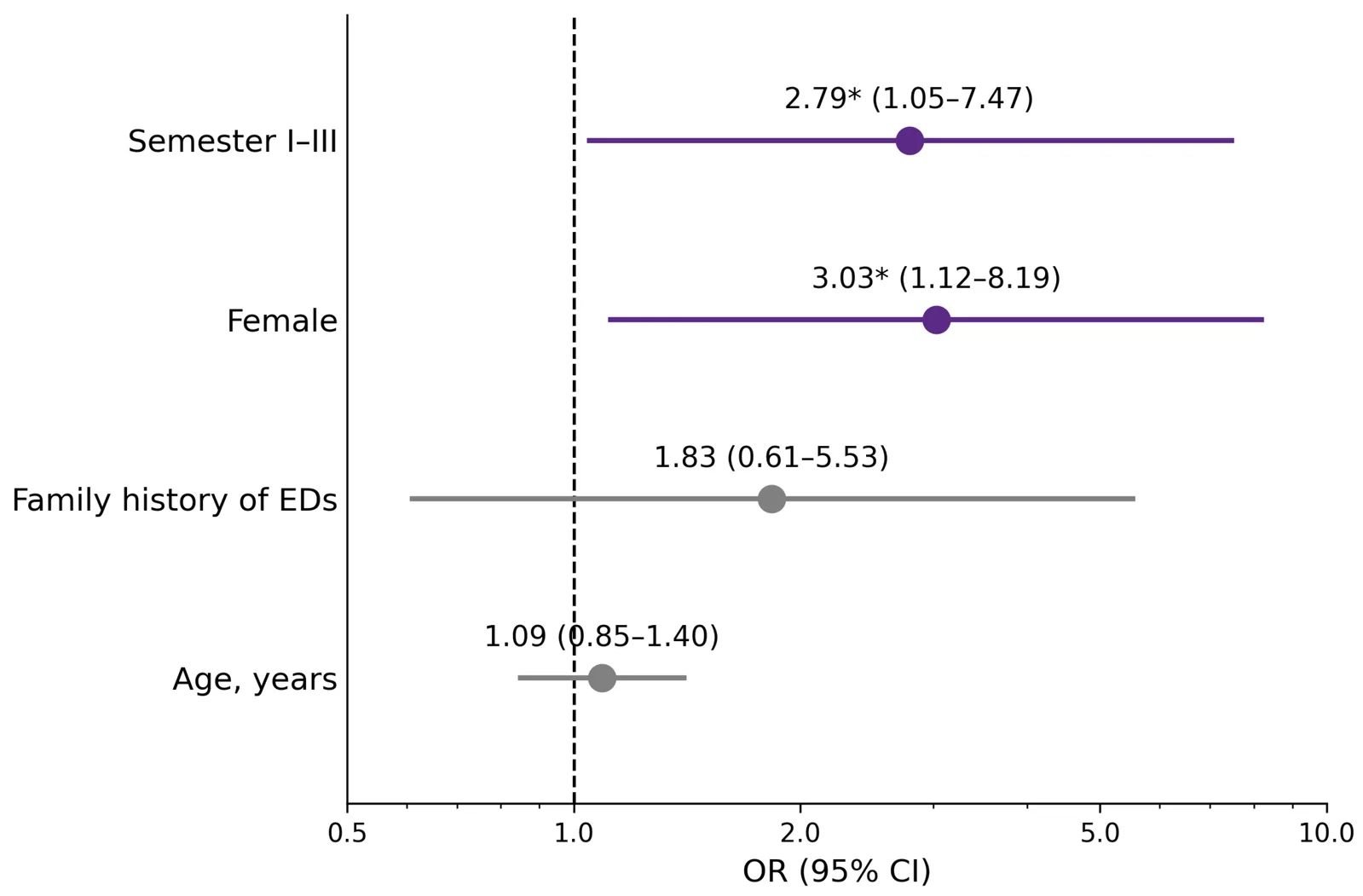
Forest plot of factors associated with high risk of EDs. Points show adjusted OR, bars show 95% CI; dashed line marks OR = 1. Purple = significant (*p* < 0.05, marked *); gray = non-significant.

**Table 1:** Sociodemographic and Clinical Characteristics of the Study Population.

Characteristic	Overall n = 167 ^1^	Risk EAT-26		*p*-Value
		Low n = 136 ^1^	High n = 31 ^1^	
Demographics				
Age, years	19.9 ± 1.8	20.0 ± 1.8	19.7 ± 1.8	0.4 ^2^
Sex				0.007 ^3^
Male	62 (37%)	57 (42%)	5 (16%)	
Female	105 (63%)	79 (58%)	26 (84%)	
Marital status				0.2 ^4^
Single	159 (95%)	131 (96%)	28 (90%)	
Free union	8 (4.8%)	5 (3.7%)	3 (9.7%)	
Semester				0.014 ^4^
I–III	43 (26%)	29 (21%)	14 (45%)	
IV–VI	79 (47%)	71 (52%)	8 (26%)	
VII–X	40 (24%)	31 (23%)	9 (29%)	
Internship	5 (3.0%)	5 (3.7%)	0 (0%)	
Socioeconomic stratum				0.2 ^4^
I–II	62 (37%)	49 (36%)	13 (42%)	
III–IV	89 (53%)	76 (56%)	13 (42%)	
V–VI	16 (9.6%)	11 (8.1%)	5 (16%)	
Lifestyle				
Living arrangement				0.3 ^4^
Family	116 (69%)	97 (71%)	19 (61%)	
Independent	22 (13%)	18 (13%)	4 (13%)	
Peer network	29 (17%)	21 (15%)	8 (26%)	
Work and study	11 (6.6%)	8 (5.9%)	3 (9.7%)	0.4 ^4^
Regular physical activity	75 (45%)	66 (49%)	9 (29%)	0.049 ^3^
Sleep hours				0.5 ^4^
Insufficient	39 (23%)	30 (22%)	9 (29%)	
Borderline	105 (63%)	88 (65%)	17 (55%)	
Recommended	23 (14%)	18 (13%)	5 (16%)	
Perceived stress				0.74 ^4^
Low	7 (4.2%)	6 (4.4%)	1 (3.2%)	
Medium	103 (62%)	86 (63%)	17 (55%)	
High	57 (34%)	44 (32%)	13 (42%)	

EAT-26: Eating Attitudes Test-26. Sleep hours: Insufficient (<5 h); Borderline (5–6 h); Recommended (>7 h). ^1^ Mean ± SD; n (%); ^2^ Welch’s two-sample *t*-test; ^3^ Pearson’s Chi-squared test; ^4^ Fisher’s exact test.

**Table 2:** Medical and Psychiatric History in Undergraduate Medical Students.

History	Overall n = 167 ^1^	Risk EAT-26		*p*-Value
		Low n = 136 ^1^	High N = 31 ^1^	
Family history of EDs	20 (12%)	14 (10%)	6 (19%)	0.212 ^2^
Psychiatric	27 (16%)	20 (15%)	7 (23%)	0.3 ^3^
Anxiety	13 (7.8%)	11 (8.1%)	2 (6.5%)	>0.9 ^2^
Depression	12 (7.2%)	9 (6.6%)	3 (9.7%)	0.5 ^2^
Bipolar disorder	1 (0.6%)	1 (0.7%)	0 (0%)	>0.9 ^2^
ADHD	1 (0.6%)	1 (0.7%)	0 (0%)	>0.9 ^2^
Hypertension	1 (0.6%)	1 (0.7%)	0 (0%)	>0.9 ^2^
Migraine	1 (0.6%)	1 (0.7%)	0 (0%)	>0.9 ^2^
Absence seizures	1 (0.6%)	1 (0.7%)	0 (0%)	>0.9 ^2^

EAT-26: Eating Attitudes Test-26; ADHD: Attention-Deficit/Hyperactivity Disorder; EDs: Eating Disorders. ^1^ n (%); ^2^ Fisher’s exact test; ^3^ Pearson’s Chi-squared test.

**Table 3:** EAT-26 Test Evaluation in Undergraduate Medical Students.

Characteristic	Overall n = 167 ^1^	Sex		*p*-Value
		Male n = 62 ^1^	Female n = 105 ^1^	
Domain				
Dieting	6.8 ± 6.7	5.0 ± 4.8	7.9 ± 7.4	0.014 ^2^
Bulimia	1.6 ± 2.4	1.5 ± 2.4	1.7 ± 2.4	0.671 ^2^
Oral Control	3.9 ± 3.2	3.0 ± 2.3	4.4 ± 3.5	0.007 ^2^
Total Score	12.4 ± 9.9	9.5 ± 7.1	14.1 ± 10.9	0.005 ^2^
EAT-26 Risk				0.007 ^3^
Low	136 (81%)	57 (92%)	79 (75%)	
High	31 (19%)	5 (8.1%)	26 (25%)	

EAT-26: Eating Attitudes Test-26. ^1^ Mean ± SD; n (%); ^2^ Mann–Whitney U test; ^3^ Pearson’s Chi-squared test. Reported means are rounded independently to one decimal place; the sum of the rounded subscale means differs slightly from the rounded total score mean due to independent rounding (unrounded subscale means sum exactly to the unrounded total mean).

**Table 4:** Factors Associated with High Risk of EDs—Pre-specified Multivariate Logistic Regression Model.

Characteristic	Univariate ^1^			Adjusted ^1^		
	OR	95% CI	*p*-Value	OR	95% CI	*p*-Value
Age, years	1.08	0.84, 1.40	0.55	1.09	0.85, 1.40	0.50
Female	3.35	1.18, 9.47	0.023	3.03	1.12, 8.19	0.029
Semester I–III	2.83	1.04, 7.69	0.041	2.79	1.05, 7.47	0.041
Family history of EDs	1.81	0.59, 5.57	0.30	1.83	0.61, 5.53	0.28

EAT-26: Eating Attitudes Test-26. ^1^ Firth’s penalized (bias-reduced) logistic regression, appropriate given the low number of high-risk events (n = 31) relative to candidate predictors. Model pre-specified a priori as sociodemographic and clinical variables with recognized relevance to eating disorder risk, rather than derived through post hoc variable selection. Events-per-variable = 7.25.

## Data Availability

The datasets generated and analyzed during the present study are not publicly available due to confidentiality considerations but are available from the corresponding author upon reasonable request.

## Supplementary Materials

The following supporting information can be downloaded at: https://www.mdpi.com/article/10.3390/healthcare14193314/s1. Table S1. Sensitivity Analysis—Full Candidate Model (Firth-Penalized, 15 Predictors).

## Abbreviations

EDs	Eating Disorders
EAT-26	Eating Attitudes Test-26
aOR	Adjusted Odds Ratio
OR	Odds Ratio
CI	Confidence Interval
SD	Standard Deviation
ADHD	Attention-Deficit/Hyperactivity Disorder
