# Supplementary material for: Prevalence and Factors Associated with the Risk of Eating Disorders Among Medical Students
Source: Healthcare (Basel). 2026 Oct 5;14(19):3314. doi: 10.3390/healthcare14193314 (PMC13665289; doi:10.3390/healthcare14193314)
Supplement: Supplementary file 1 [file healthcare-14-03314-s001.zip › healthcare-4567810-supplementary.pdf]

Article: Prevalence and factors associated with the risk of eating disorders among medical students

Supplementary Table S1. Sensitivity Analysis — Full Candidate Model (Firth-Penalized, 15 Predictors)

Characteristic	Firth OR	95% CI	p-value
Age, years	1.04	0.78, 1.38	0.79
Female	2.52	0.88, 7.28	0.087
Free union	5.41	1.04, 28.07	0.045
Semester I–III	4.64	1.46, 14.68	0.009
Semester VII–X/Internship	1.58	0.48, 5.14	0.45
Stratum III–IV	0.99	0.39, 2.55	0.99
Stratum V–VI	2.30	0.52, 10.28	0.27
Regular physical activity	0.84	0.33, 2.16	0.72
Borderline sleep hours	0.37	0.13, 1.08	0.069
Recommended sleep hours	0.46	0.10, 2.09	0.31
Low perceived stress	1.41	0.15, 12.76	0.76
High perceived stress	2.36	0.94, 5.92	0.067
Personal psychiatric history	1.31	0.32, 5.42	0.71
Family history of EDs	1.38	0.43, 4.42	0.59
Depression	0.89	0.14, 5.60	0.90

Firth-penalized logistic regression across all 15 originally candidate predictors (EPV = 1.93), provided to demonstrate the sensitivity of estimates to model specification. Reference categories: male sex; single marital status; semester IV–VI; socioeconomic stratum I–II; no regular physical activity; insufficient sleep; medium perceived stress; no personal or family psychiatric history; no depression. Semester I–III remains robust across specifications; the female-sex association attenuates and loses significance when the model is saturated with covariates, illustrating the estimate instability raised in peer review. As previously noted, "Personal psychiatric history" and "Depression" are not independent (depression contributes to the aggregate psychiatric-history variable in Table 2); their joint inclusion here is retained only to reproduce the originally submitted candidate set for comparison and should not be interpreted as two independent risk factors.
